# Antimicrobial Activity of 3D-Printed Acrylonitrile Butadiene Styrene (ABS) Polymer-Coated with Silver Nanoparticles

**DOI:** 10.3390/ma14247681

**Published:** 2021-12-13

**Authors:** Isabel Tse, Atishay Jay, Ina Na, Sean Murphy, Nereida Niño-Martínez, Gabriel Alejandro Martínez-Castañon, Jamie Magrill, Horacio Bach

**Affiliations:** 1 Division of Infectious Diseases, Faculty of Medicine, University of British Columbia, Vancouver, BC V6H3Z6, Canada; isabel-tse@hotmail.com; 2 School of Biomedical Engineering, University of British Columbia, Vancouver, BC V6H3Z6, Canada; atishay1@student.ubc.ca; 3 DECAP Research & Development Inc., Richmond, BC V7C2A6, Canada; inana96@gmail.com (I.N.); jamie@decap.ca (J.M.); 4 Centre for High-Throughput Phenogenomics, Faculty of Dentistry, University of British Columbia, Vancouver, BC V6H3Z6, Canada; sean.murphy@dentistry.ubc.ca; 5 Facultad de Ciencias, Universidad Autónoma de San Luis Potosí, San Luis Potosí 78000, Mexico; nereyda.nino@uaslp.mx; 6 Facultad de Estomatología, Universidad Autónoma de San Luis Potosí, San Luis Potosí 78000, Mexico; mtzcastanon@fciencias.uaslp.mx

**Keywords:** 3D printing, antibacterial, antifungal, multi-drug resistant, silver nanoparticles, acrylonitrile butadiene styrene

## Abstract

Medical devices with antimicrobial properties are a potential long-term solution to the high rate of multi-drug-resistant healthcare-associated infections. Silver nanoparticles (AgNPs) are an established agent for effectively eliminating a wide range of microbial strains. AgNPs have been commonly incorporated into traditional plastic materials; however, recently, there has been increased interest in using AgNPs combined with 3D-printing technology for medical devices due to the accessibility and customizability of 3D-printed products. This study reports a novel method of utilizing acetone to partially dissolve 3D-printed polymer acrylonitrile butadiene styrene (ABS) plastic to attach a layer of AgNPs. The antimicrobial properties of this AgNP-coated surface were tested against several microbial strains prevalent in healthcare-associated infections. AgNP-coated ABS (AgNP-ABS) plastic demonstrated significant elimination of viable bacteria within 4 h for all tested bacterial species (*Acinetobacter baumannii*, non-pathogenic and pathogenic *Escherichia coli*, *Pseudomonas aeruginosa*, and methicillin-resistant *Staphylococcus aureus*) and within 19 h for the tested fungus *Candida albicans*. The longevity of adhesion of AgNPs to the ABS plastic was assessed by checking antibacterial activity against *A. baumannii* after repeat use cycles. AgNP-ABS plastic showed decreased antibacterial efficacy with repeated use but maintained the ability to eliminate microbes within 3 h for up to eight use cycles. The AgNP-coated ABS plastic showed efficacy as an antimicrobial surface, and future studies will consider its applicability in the production of medical devices.

## 1. Introduction

Silver nanoparticles (AgNPs) have been reported to have antimicrobial effects against various strains of bacteria, fungi, and viruses [1,2,3,4]. These antimicrobial effects are thought to result from the steady release of Ag^+^ from AgNPs into the environment. Ag^+^ is subsequently free to adhere to the cell membranes of microbes and contributes to cell death through unbalancing cell membrane structure. In addition, Ag^+^ might generate reactive oxygen species (ROS), protein inactivation, and lipid peroxidation [1,5]. Furthermore, Ag^+^ interactions result in structural condensation of DNA, preventing DNA replication and cell reproduction [6].

In the last eight decades, an increasing number of pathogenic bacteria have developed resistance to commonly used antibiotics [7,8,9]. The emergence of multi-drug resistant pathogens has contributed to a growing interest in using AgNPs as an alternative antimicrobial agent. AgNPs have demonstrated antimicrobial efficacy against many microorganisms, including multi-drug resistant pathogens [10,11,12,13]. The appeal of using AgNPs can also be attributed to their ability to eliminate microorganisms at low concentrations that are non-cytotoxic to mammalian cells [14,15]. For these reasons, the antimicrobial properties of AgNPs have been exploited for use in a wide variety of applications such as water purification systems, commercial food containers, and medical devices [16,17,18,19]. AgNPs have also commonly been incorporated into traditional plastic materials like polyethylene (PE), polyvinyl chloride (PVC), and polyurethane for commercial usage [19,20,21].

The most commonly used 3D-printing process is fused deposition modeling, which involves the deposition of thermoplastic material filaments to create 3D objects. The nozzle of the 3D printer melts plastic filaments and moves in two dimensions to extrude the material strand by strand in the X-Y plane, then moves vertically to deposit another layer. Because of the directional way 3D printers deposit plastic, 3D-printed parts have structural properties unique from traditional plastic manufacturing parts. For example, channels of air pockets are created between the strands of extruded plastic, and the porous structure of 3D prints could potentially contribute to the accumulation of microbes [22,23].

The advent of 3D-printed plastic materials has increased interest in applying AgNPs in combination with 3D-printed plastics. However, the evidence is absent in the literature for the applicability of AgNPs with 3D-printed plastics. In addition, 3D-printed plastics do not have the same properties as traditionally manufactured plastics, and thus they should be evaluated independently for efficacy as an antimicrobial surface.

Acrylonitrile butadiene styrene (ABS) is a typical thermoplastic copolymer used in 3D printing as it produces durable, rigid, and chemically resistant plastic at a low cost [24]. However, ABS plastics can be dissolved at room temperature (25 °C) by acetone, and acetone vapour smoothing is a commonly used post-extrusion technique to finish the ABS surface prints with minimal alteration of the print design [25,26]. Notably, soaking ABS prints in acetone solution has also been established as a technique to decrease the porosity of the plastic structure as the dissolved ABS tends to redeposit into the air gaps [27].

Recent studies investigated methods to incorporate nanoparticles into 3D-printing materials focused on mixing AgNPs with polymers before filament extrusion of objects [28,29]. While these approaches have proven successful in combining antimicrobial properties to 3D prints, this paper presents an alternative methodology that entails coating finished 3D-printed ABS objects with an antimicrobial AgNP layer. This technique has comparatively more streamlined steps and utilizes inexpensive and accessible materials.

The present study introduces a novel method of coating ABS plastics with AgNPs to create an antibacterial surface coating. We utilized partial dissolution of ABS by acetone to deposit AgNPs onto the surface of ABS plastics. Rather than incorporating NPs in a plastic-polymer suspension before extrusion and hardening, we opted for the dispersal of NPs only on the outer surface of ABS plastics as a more cost-effective approach. Thus, only the contact layer of the material would have antimicrobial activity as a potential release of Ag^+^ to the environment, contributing to bactericidal and fungicidal activity, leading to a reduction of the overall amount of AgNPs required to achieve antimicrobial effects.

To evaluate our novel method for AgNP deposition to create an antimicrobial surface coating, we exposed AgNP-coated ABS plastics (AgNP-ABS) to a suite of bacteria and fungi commonly associated with healthcare infections compared to uncoated, acetone-smoothed ABS plastics (control-ABS). We further assessed the longevity of adhesion of AgNPs to ABS plastics with usage over time.

## 2. Materials and Methods

### 2.1. Preparation of AgNPs

AgNPs were prepared by mixing 100 mL of an AgNO_3_ solution (0.01 m) with 10 mL of a 0.01 g/mL tannic acid solution at room temperature and under magnetic stirring. After the mixing was completed (about 20 s), the pH was raised to 10.0 using NaOH (3.0 m). The reaction was transferred to a plastic container after stirring for another 20 min. The VIS-NIR absorption spectrum was obtained using a CHEMUSB4-VIS-NIR spectrophotometer (Ocean Optics, Orlando, FL, USA). Transmission electron microscope (TEM) images were obtained with a JEOL JEM-1230 microscope (JEOL, Tokyo, Japan) operated at an acceleration voltage of 100 kV. Hydrodynamic diameter and zeta potential were measured using dynamic light scattering (DLS) with a Zetasizer Nano ZS (Malvern Instruments, Malvern, United Kingdom) operating with a He-Ne laser at a wavelength of 633 nm and a detection angle of 90 °C at 25 °C.

### 2.2. Disk Production

The production of the disks was performed with yellow ABS plastics (Spool 3D, Ontario, ON, Canada). The tolerance was +/− 0.04 mm with an optimum extrusion temperature of 220–260 °C. Quantitative tests of the 3D printer showed the optimum temperature for the desired printing speeds was 240 °C, which was used for producing these disks. The filament had a maximum melt flow rate of 24 g/10 min. The tensile strength of the disks was 40 MPa, and the elongation at break was 12%. The flexural strength was 64 MPa, and the flexural modulus was 2200 MPa. The Notched Izod impact strength was 210 J/m. The heat distortion temperature was slightly lower than the average blend of ABS, at 85 °C.

### 2.3. Printer Attributes

The 3D printer was equipped with a 0.4 mm diameter copper nozzle. Copper was chosen over brass for this application due to its better heat conductivity, which allowed printing the disks at a lower temperature while maintaining adequate flow and low extrusion path resistance. This, in turn, allowed us to use less fan speed, resulting in more substantial pieces. Furthermore, the copper nozzle enables higher flow rates, allowing for faster printing and lower lead times.

The hot end used on this device was the Dragon HF (Triangle Labs, Carson City, NV, USA). The HF variant has ultra-thin walls for the heat-break, preventing heat-creep defects. It also has a higher melting rate than the standard variant, allowing for high-speed 3D printing. The extruder is a direct drive and based on hardened steel BMG parts with a small Bowden guide tube within the extruder allowing for printing flexible filaments without tangling.

The printer has a chassis made of aluminum extrusions, with two rails holding up a 350 mm × 350 mm × 400 mm build area. The printer is fully enclosed, and the chamber reached 50 °C, allowing for dimensionally accurate ABS/ASA printing.

### 2.4. Plastic Disc Coating

**Figure 1:**
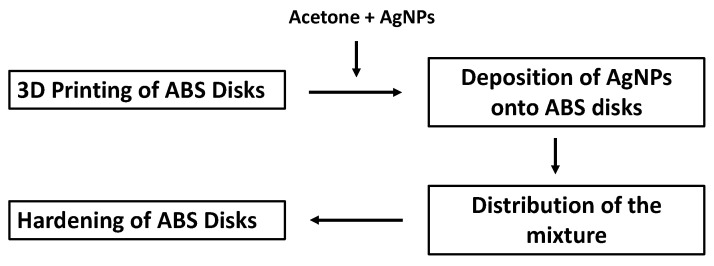
Process of embedding AgNPs on 3D-printed ABS disks.

ABS plastic discs were 3D printed with uneven rectilinear surfaces. These discs were smoothed by submerging the discs into 100% acetone for 30 s and pressing the surface onto a clean glass surface. The discs were allowed to reharden overnight, and a mixture of 10 µL (0.1 µg) of AgNPs and 80 µL 100% acetone was applied to the ABS plastic discs’ surface. A sterile plastic inoculation loop was used to distribute the NP-acetone mixture evenly on the surface for 30 s until the surface absorbed the majority of the liquid suspension. After drying for 3 h, the same NP application procedure was repeated on the opposite side of the ABS plastic discs. The surface of each coated ABS plastic disc used for testing had an estimated AgNP concentration of 19.72 ng/cm^2^. A scheme showing the different steps in the process is detailed in Figure 1.

### 2.5. SEM Analysis of the Disks

Electron microscopy was performed in the Centre for High-Throughput Phenogenomics (University of British Columbia, Vancouver, BC, Canada). Samples were affixed to aluminum SEM stubs with adhesive carbon tape for imaging. The samples were made conductive utilizing a 7 nm thick sputtered iridium coating. Sputtering was performed using a Leica EM MED020 coater (Leica Microsystems, Wetzlar, Germany). The sputtered film thickness was monitored during deposition using a quartz crystal microbalance. All imaging was acquired with an FEI Helios Nanolab 650 FIB-SEM (Hillsboro, OR, USA). For imaging with the backscattered electron detector, a second sample with a carbon coating was prepared; 15 nm of carbon were evaporated onto the sample surface using a Leica EM MED020 coater.

### 2.6. Evaluation of Antimicrobial Activity

Antimicrobial activity of AgNP-ABS discs was assessed against a non-pathogenic bacterial strain of *Escherichia coli* (DH5α), the fungal pathogen *Candida albicans* (provided by Vancouver General Hospital, Vancouver, BC, Canada), and four different multidrug-resistant strains of the following pathogenic bacteria *Acinetobacter baumannii*, pathogenic *E. coli*, *Pseudomonas aeruginosa*, and *Staphylococcus aureus*. The characterization of these strains was described previously [30]. A bacterial suspension (200 µL) ranging from 4 × 10^4^–4 × 10^5^ CFU/mL was distributed onto the surface of AgNP-ABS and control-ABS discs. The exact concentration of viable cells for each strain post-application was determined using serial plating. The applied microorganisms were allowed to interact with the discs at room temperature. A recovery test was performed on untreated discs to confirm an equal number of microorganisms were recovered from the discs. Samples were collected at the time points: 0.5, 1, 2, and 3 h for *A. baumannii*, non-pathogenic and pathogenic *E. coli*, and *P. aeruginosa*; 1, 2, 3, and 4 h for *S. aureus*, and 1, 2, 3, 4, 19, and 24 h for *C. albicans*. After experimental time points were reached, the microorganisms were released from the plastic discs by shaking with 1 mL of sterile PBS at room temperature at 150 rpm for 30 min. Samples were taken from the supernatant and then serially diluted for inoculation on Mueller-Hinton agar plates (B & D, Franklin Lakes, NJ, USA). The number of viable cells was counted after 18 h overnight incubation at 37 °C. *C. albicans* samples were inoculated on Sabouraud-Dextrose agar plates (B & D, Franklin Lakes, NJ, USA) and incubated at 30 °C for 24 h.

### 2.7. Longevity of Adhesion of AgNP Coatings

AgNP coating adhesion longevity was evaluated by exposing NP-free ABS (control-ABS) and AgNP-ABS discs to the strain *A. baumannii* used as a model and conducting a series of use cycles. AgNPs were only applied before the first use cycle. Each use cycle consisted of exposure of *A. baumannii* to AgNP-ABS discs followed by an incubation of 0.5, 1, 2, or 3 h, followed by a wash of 1 mL of sterile PBS for 30 min at 150 rpm, measurement of bacterial growth, and then a second wash in fresh sterile PBS for 20 min. Discs were allowed to dry before the next use cycle. Each disc was treated for 10 use cycles to determine the longevity of antimicrobial activity of AgNP-ABS discs.

## 3. Results

### 3.1. Characterization of AgNPs

**Figure 2:**
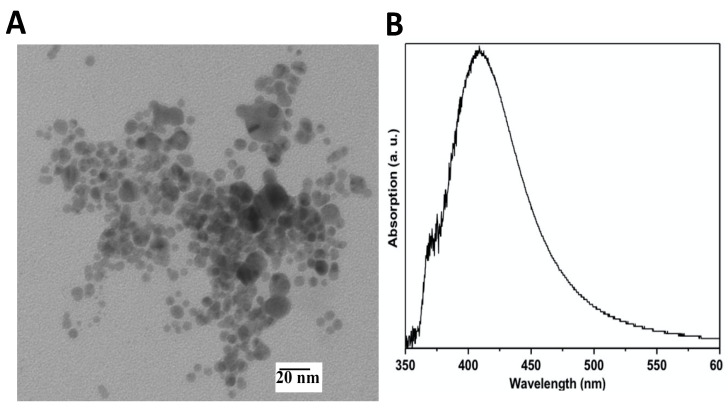

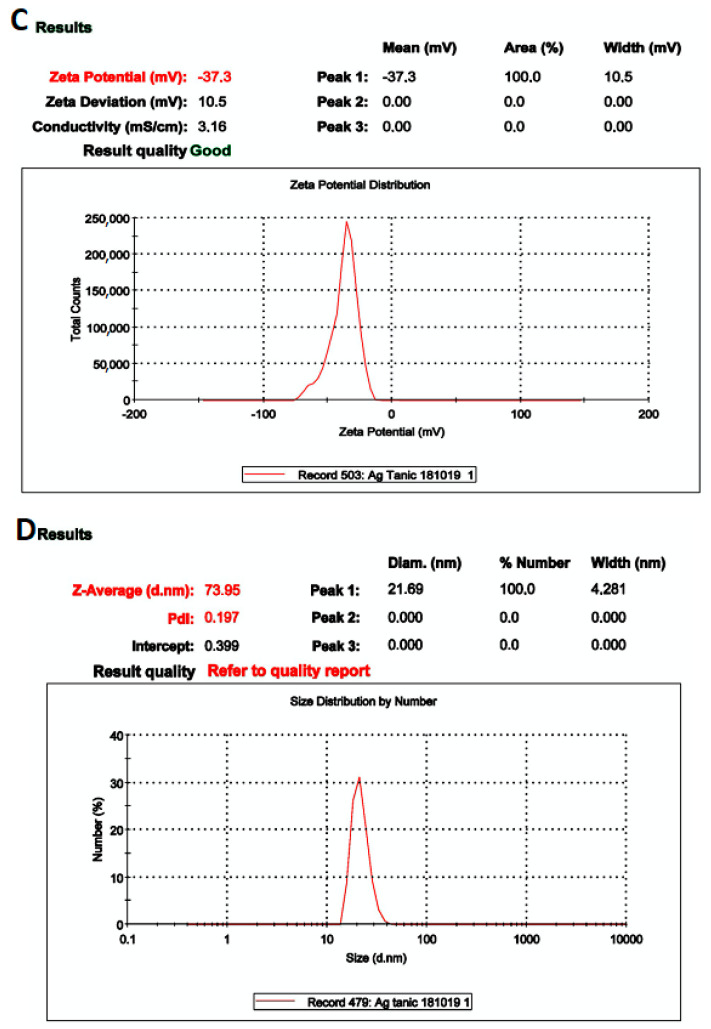
Characterization of AgNPs. (**A**) TEM image, (**B**) Surface plasmonic resonance is located at 412 nm, (**C**) DLS showing the zeta potential, and (**D**) the hydrodynamic diameter.

The prepared AgNPs were obtained as a stable dispersion. The AgNPs showed a spherical morphology (Figure 2A) with a surface plasmon resonance at 415 nm (Figure 2B). The zeta potential value for these NPs was −37.1 mV (Figure 2C), whereas the hydrodynamic diameter was found to be 21.7 ± 2.1 nm (Figure 2D).

### 3.2. SEM Analysis of the ABS before and after AgNP Coating

**Figure 3:**
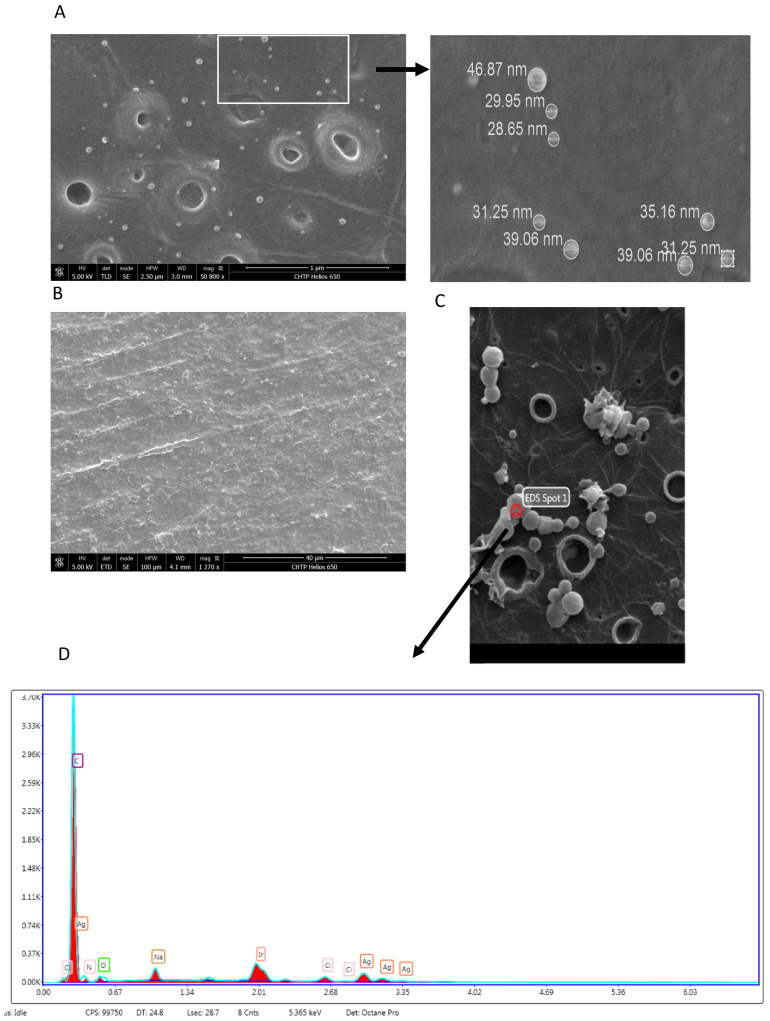
Analysis of AgNPs on the ABS disks.Disks were analyzed by SEM to determine the AgNPs distribution. The SEM analysis was performed according to the Materials and Methods section. (**A**) Disks were treated with AgNPs, and the sizes of the AgNPs are shown in the inset. (**B**) Untreated disks (control). (**C**) Selected area of the treated disk used for X-ray spectroscopy. (**D**) Analysis of elements detected by SEM-EDS.

SEM analysis showed that the AgNPs were embedded on the ABS disks, as shown in Figure 3. Images showed that AgNPs were embedded into the disks (Figure 3A) but not in the untreated control (Figure 3B).

### 3.3. AgNP-Coated 3D-Printed ABS Demonstrated Antimicrobial Activity

**Figure 4:**
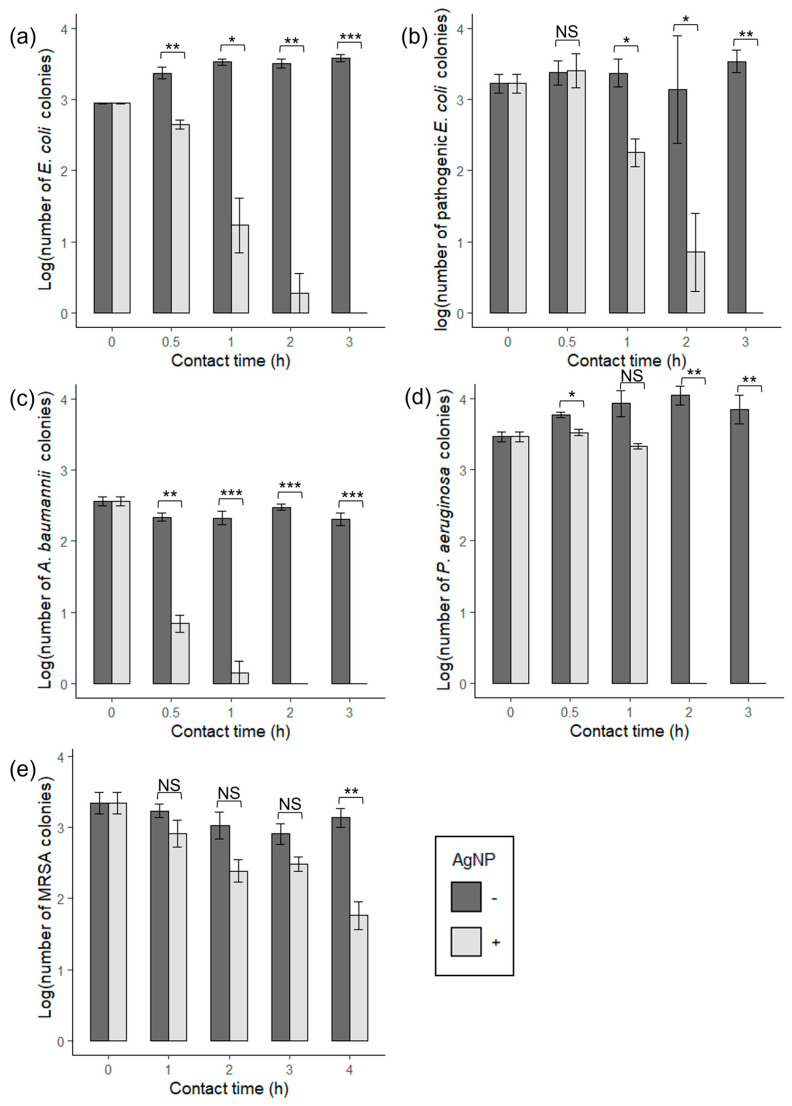
Antibacterial activity of AgNPs impregnated on ABS 3D-printed discs.The antibacterial activity of the discs was assessed on plastics and according to the materials and methods section. Results are expressed as the log number of viable bacterial colonies (CFU/mL) as a function of the contact time in hours. (**a**) non-pathogenic *E. coli*, (**b**) pathogenic *E. coli*, (**c**) *A. baumannii*, (**d**) *P*. *aeruginosa*, and (**e**) MRSA with and without AgNP coating. Shown are the means ± SE of three independent experiments. A *t*-test was used for statistical analysis, * *p* ≤ 0.05, ** *p* ≤ 0.01, *** *p* ≤ 0.001. NS, no significance.

To assess the antimicrobial properties of AgNP-coated ABS plastic, we examined changes in viability of a suite of microorganisms commonly associated with healthcare infections as a function of contact time. AgNP-ABS discs display a remarkable efficacy in killing microorganisms compared to control-ABS discs, demonstrating significant bactericidal activity against all tested bacterial strains (Figure 4). These results also showed that with a longer contact time on the AgNP-coated discs with the pathogens, a decrease in the CFU/mL was observed. No bacterial survival was observed after 3 h and 4 h contact time with AgNP-ABS discs for Gram-negative and -positive strains (Figure 4). In summary, we demonstrated that a surface coating of ABS plastic with an AgNP concentration of 19.72 ng/cm^2^ is sufficient to kill bacterial cells after 3 h contact.

Among the Gram-negative species, *A. baumannii* showed the most rapid reduction in CFU count with a log reduction > 3.0 after 0.5 h exposure with a total killing after 2 h exposure. In the case of *P. aeruginosa*, a total killing was measured after 3 h of contact time. Non-pathogenic and pathogenic *E. coli* showed similar loss of viability patterns as a function of contact time, with complete loss of viability after 3 h of contact time.

**Figure 5:**
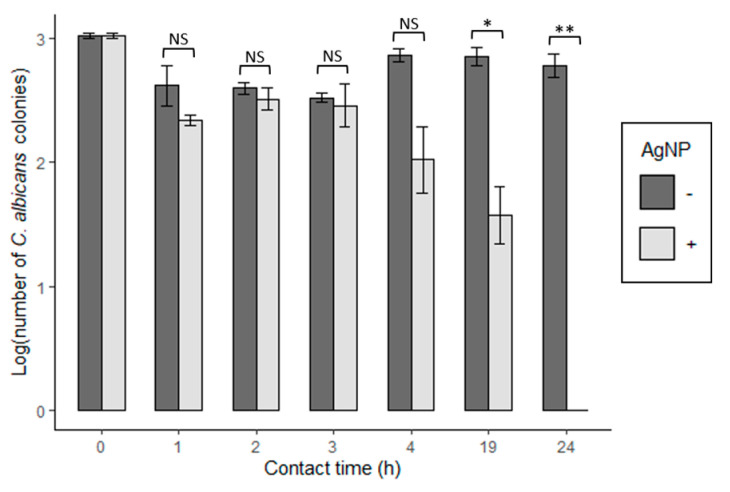
Antifungal activity of AgNPs impregnated on ABS 3D-printed discs.The antifungal activity of the discs on *C. albicans* was assessed on plastics and according to the materials and methods section. Results are expressed as the log number of viable microbial colonies (CFU/mL) as a function of the contact time (h) with and without AgNP coating. Shown are the means ±SE of three independent experiments. A *t*-test was used for statistical analysis, * *p* ≤ 0.05, ** *p* ≤ 0.01. NS, no significance.

Regarding the antifungal activity AgNP-coated of the discs, it seemed that the AgNPs could not entirely reduce the fungal viability of *C. albicans* within the same range of contact time (4 h) as seen with the bacterial strains. However, after 19 h of contact time, we eventually observed the complete killing of *C. albicans* (Figure 5).

### 3.4. AgNPs Show Reduced Effectiveness after Usage Testing

**Figure 6:**
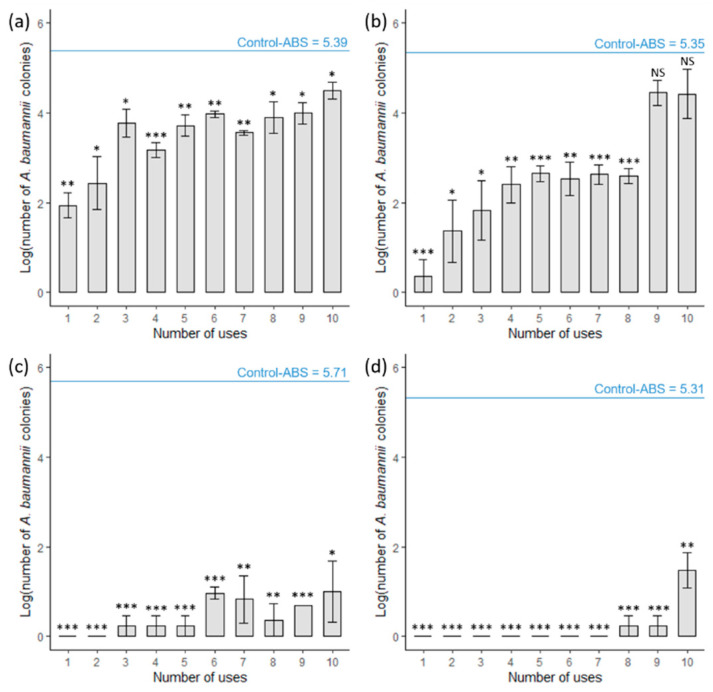
Antibacterial activity of ABS 3D-printed discs as a function of use cycles. The antibacterial activity of the discs on *A. baumannii* was assessed on plastics and according to the materials and methods section. Results are expressed as the log number of viable bacterial colonies (CFU/mL) as a function of the contact time (h) with and without AgNP coating. Bacteria were in contact with discs for (**a**) 0.5 h, (**b**) 1 h, (**c**) 2 h, and (**d**) 3 h. The log number of bacteria retrieved from the untreated control is represented by the blue line used as a reference. Shown are the means ± SE of three independent experiments. A *t*-test was used for statistical analysis, * *p* ≤ 0.05, ** *p* ≤ 0.01, *** *p* ≤ 0.001. NS, no significance.

We interrogated the longevity of adhesion of our AgNP coating by observing the change in the number of *A. baumannii* CFU before AgNP application, during, and after 10 use cycles. As a baseline, we demonstrated that AgNP-ABS discs exposed once to *A. baumannii* showed a rapid reduction in bacterial activity with total loss of viability after 2 h (Figure 6). However, after three use cycles, we found that 2 h of contact time was no longer sufficient to abolish bacterial activity entirely, but 3 h of contact time was adequate for complete loss of viability until the eighth use cycle (Figure 6). Overall, we observed that AgNP-ABS discs across all contact times decreased their effectiveness after 10 use cycles.

## 4. Discussion

This study demonstrated that an AgNP coating on standard ABS 3D-printed plastic displayed antimicrobial activity against a suite of bacteria and a fungus commonly related to healthcare-associated infections. This AgNP-mediated loss of bacterial viability depended on contact time and degree of usage (Figure 4 and Figure 5). Antibacterial testing of the AgNP-ABS plastic was carried out on both Gram-negative and -positive bacterial species. AgNP-coated ABS plastic demonstrated higher elimination rates for Gram-negative bacteria than Gram-positive bacteria. For example, log reductions ranging between 2.5 to 4 CFU/mL were measured when the Gram-negative strains were exposed to the treated disks for 2–3 h (Figure 4a–d).

In contrast, around 1 log reduction was observed when the Gram-positive MRSA strain was exposed to the treated disks for 4 h. The differences in the rate of loss of viability between the Gram groups are likely due to differences in their cell wall structure. Gram-negative bacteria have an additional protective outer membrane compared to Gram-positive bacteria that prevents many molecules (e.g., antibiotics) from passing through the cell. Moreover, the cell wall of Gram-positive bacteria has a thicker layer of peptidoglycan than Gram-negative bacteria [1]. While all the mechanistic antibacterial actions of AgNPs have not been fully elucidated, it has been reported that AgNP permeates through this outer membrane and creates pit-like structural damage to the cell wall [30].

Moreover, AgNPs have been shown to damage the peptidoglycan cell wall of Gram-positive bacteria in a similar fashion [31]. Therefore, the presence of this outer membrane could be irrelevant to the antibacterial efficacy and somewhat dependent on the thickness of the cell wall layer. The thinner peptidoglycan layer could be a reason why AgNPs are more effective at killing Gram-negative bacteria.

Aligning with our results, Gram-positive bacteria have been reported in the literature to be more resistant to AgNP-inhibition than Gram-negative bacteria [32,33]. While AgNPs have generally shown a more significant bactericidal effect on Gram-negative bacteria than Gram-positive bacteria in literature, this may not be the sole factor in determining a strain’s susceptibility to AgNPs. For example, it has been demonstrated that the antimicrobial efficiency of AgNPs can vary within different strains of *E. coli*, suggesting that it is influenced by other factors than simply the structure of the bacterial membrane [34]. This observation aligns with our findings that our tested pathogenic *E. coli* strain shows greater resistance to the bactericidal effects of AgNP than the non-pathogenic *E. coli* strain (Figure 2A,B). For instance, after 30 min of contact time, a significant reduction in colonies was only observed in non-pathogenic *E. coli*.

Moreover, bactericidal activity is dependent on the shape of the nanoparticle [35]. For example, truncated triangular nanoplates show greater efficiency in inhibition of bacterial activity than spherical and rod-shaped AgNPs. The surface interaction between AgNPs and cell membranes may have a role in inhibition rates. However, the specific estimation of how the surface area of NPs relates to the AgNP mechanism of action has yet to be explored. Notably, all of the testing in our study was done solely on AgNP-ABS created with one type of AgNP. We speculate that our presented results of the relative antimicrobial effects between different strains of bacteria may differ if another shaped AgNP was used. Our main intent was to demonstrate that our coating technique can harness the antimicrobial properties of AgNPs onto the surface of 3D-printed ABS.

In the present study, *C. albicans* demonstrated a higher degree of resistance to the antimicrobial effects of AgNP. For example, with a contact time of 4 h, we did not observe a significant reduction of fungal viability, unlike with our bacterial strains. However, with a longer contact time of 24 h, AgNP-ABS plastic eliminated all viable *C. albicans* cells (3 log reduction) (Figure 5).

The antifungal activity of NPs against fungal strains has not been completely elucidated. For example, previous studies reported that the antifungal activity against *C. albicans* depended on the size and the element used in the AgNP fabrication, the fungal strain, ROS production, etc. For instance, a killing activity against *C. albicans* was observed with NP sizing 5 nm, whereas a size of 100 nm showed no activity [36]. In the same report, ROS production increased after exposure to 5 nm AgNPs to *C. albicans*. Still, no ROS increase was measured when the same AgNPs were exposed to the yeast *Saccharomyces cerevisiae* used as control [36]. In addition, other extracellular and intracellular interactions of AgNPs with *C. albicans* cells might explain the antifungal activity. For instance, the net charge of the AgNPs can interact with the fungal cell wall, causing cell lysis as a result of an unbalance in the electrostatic homeostasis of the wall [37], leading to its destruction [33,38,39].

The difference in resistance we observed between fungal and bacterial strains to AgNPs may result from differences between fungal and bacterial cell walls composed of chitin and beta-glucans as opposed to peptidoglycans, which may afford additional protection from AgNP action. Despite the difference in action time, the AgNP-ABS plastic ultimately demonstrated antimicrobial activity with each tested bacterial and fungal strains.

The generation of higher levels of intracellular ROS surpassing endogenous production due to cellular metabolism is a known theme in the toxicity of AgNPs. For example, ROS are toxic molecules reacting with multiple biomolecules, such as proteins, DNA, and lipids, leading to the cell’s death [12,40]. Furthermore, regardless of the internalization of the AgNPs, Ag^+^ can be released from the NPs and react with cysteines, thiols, and indole groups, leading to a loss of enzymatic activity in the cell and blocking vital metabolic functions [1,37,41].

These results indicate that our novel coating standard ABS 3D-printed plastic with AgNPs is a reliable technique to create an antimicrobial surface. The combination of acetone and AgNPs in solution successfully deposited AgNPs onto the standard ABS 3D-printed plastic surface without disrupting the AgNPs’ antimicrobial effects. Despite the porosity of ABS 3D-printed structures, microbes can be eliminated from the surface of ABS plastics. The treatment of acetone on the ABS plastic may have also aided in decreasing the porosity of the AgNP-ABS discs [27].

We also report good durability of AgNP attachment to ABS plastic after using our cycle modeling experiments (Figure 6). The decrease in AgNP antimicrobial activity with usage is likely due to the slow leeching of Ag^+^ ions off the ABS plastic after contact with aqueous solutions. AgNP leeching is difficult to avoid entirely and occurred in other reported methods of AgNP application [17,18,42].

The method of acetone smoothing described here allows for the continued re-application of NPs without reprinting the whole plastic part. Future studies may consider the applicability of our AgNP-coated ABS plastics for use in medical devices and equipment production.

## 5. Conclusions

In conclusion, we proposed a novel approach to applying an AgNP coating to ABS plastics. This methodology exploits the highly reactive reaction of ABS plastic to acetone. The partial dissolution of the surface of printed objects allows AgNPs to be embedded into the outer layer of the polymer matrix. The resulting AgNP-ABS plastic demonstrates effective antimicrobial activity against common multi-drug resistant bacterial and fungal species. This antimicrobial property also shows reasonable durability with continued aqueous contact. These findings point towards the plausible usage of our presented technique to create antimicrobial 3D prints.

Moreover, the application of 3D printing is attractive because it allows for on-demand customized production and low volume manufacturing with minimal costs. Aligning with this intent, our proposed technique of AgNP incorporation involves minimal steps and low-cost accessible materials. For this reason, it is a feasible option for adding antimicrobial properties to 3D products for industrial usage, but more importantly, for independent purposes. The simple application of an AgNP-acetone suspension can significantly increase the value of 3D-printed products, especially those used in the healthcare sector and contact with a wide range of microbes.

## Data Availability

All the data used in this study are available.
